# Nonribosomal Peptide Synthetases in Animals

**DOI:** 10.3390/genes14091741

**Published:** 2023-08-30

**Authors:** Wouter Suring, Dylan Hoogduin, Giang Le Ngoc, Abraham Brouwer, Nico M. van Straalen, Dick Roelofs

**Affiliations:** 1 A-LIFE Ecology and Evolution, Faculty of Science, Vrije Universiteit Amsterdam, De Boelelaan 1087, 1081 HV Amsterdam, The Netherlands; 2 Department of Academy Technology & Innovation, NHL Stenden University of Applied Sciences, Rengerslaan 8-10, 8917 DD Leeuwarden, The Netherlands; 3 Biomedical Primate Research Centre, Lange Kleiweg 161, 2282 GJ Rijswijk, The Netherlands; 4 BioDetection Systems, Science Park 406, 1098 XH Amsterdam, The Netherlands; 5 Keygene N.V., Agro Business Park 90, 6708 PW Wageningen, The Netherlands

**Keywords:** NRPS, nonribosomal, peptide, synthetase, animals, Metazoa, distribution, evolution, phylogeny, beta-lactam

## Abstract

Nonribosomal peptide synthetases (NRPSs) are a class of cytosolic enzymes that synthesize a range of bio-active secondary metabolites including antibiotics and siderophores. They are widespread among both prokaryotes and eukaryotes but are considered rare among animals. Recently, several novel NRPS genes have been described in nematodes, schistosomes, and arthropods, which led us to investigate how prevalent NRPS genes are in the animal kingdom. We screened 1059 sequenced animal genomes and showed that NRPSs were present in 7 out of the 19 phyla analyzed. A phylogenetic analysis showed that the identified NRPSs form clades distinct from other adenylate-forming enzymes that contain similar domains such as fatty acid synthases. NRPSs show a remarkably scattered distribution over the animal kingdom. They are especially abundant in rotifers and nematodes. In rotifers, we found a large variety of domain architectures and predicted substrates. In the nematode *Plectus sambesii*, we identified the beta-lactam biosynthesis genes L-δ-(α-aminoadipoyl)-L-cysteinyl-D-valine synthetase, isopenicillin N synthase, and deacetoxycephalosporin C synthase that catalyze the formation of beta-lactam antibiotics in fungi and bacteria. These genes are also present in several species of Collembola, but not in other hexapods analyzed so far. In conclusion, our survey showed that NRPS genes are more abundant and widespread in animals than previously known.

## 1. Introduction

Nonribosomal peptide synthetases (NRPSs) synthesize a range of peptide products with a wide spectrum of biological functions including antibiotic and siderophore activities. They are used in industrial biotechnology to produce various pharmaceuticals such as cytostatics and immunosuppressants. NRPSs are widespread among both prokaryotes and eukaryotes, but they have only been identified in a limited number of animal species [1,2,3,4]. Recently, Shou et al. (2016) showed that the nematode *Caenorhabditis elegans* uses two NRPSs to produce the compound nemamide that promotes larval survival [2]. Homologs of these NRPSs exist in over twenty nematode species [2,5]. Furthermore, in schistosomes (Trematoda, Platyhelminthes), an NRPS is involved in the production of a male factor promoting female development [4]. In springtails (Collembola, Arthropoda), we identified the NRPS L-δ-(α-aminoadipoyl)-L-cysteinyl-D-valine synthetase that is known to catalyze the first step in beta-lactam antibiotic biosynthesis [3]. It is part of a beta-lactam biosynthesis gene cluster and is suggested to be present in over 60% of springtail families, especially in soil-living species [6]. These findings primed us to investigate how widespread NRPSs are in the animal kingdom as a whole.

NRPSs can synthesize a large range of compounds due to their modular architecture. Each module adds one amino acid to the peptide that is produced in the cytosol (nonribosomally). The enzymatic center typically consists of three domains. The adenylation domain (A domain) activates the aminoacyl substrate before transferring it to a pantetheine cofactor that is covalently attached to the peptide carrier protein domain (PCP domain) of the NRPS [7]. Next, the condensation domain (C domain) catalyzes the formation of a peptide bond between the amino acid substrates of adjacent modules. NRPS enzymes can contain many modules, resulting in a great variety of possible peptide products [8]. In addition, modules can incorporate nonproteinogenic amino acids (e.g., in the case of penicillin biosynthesis). Furthermore, NRPSs can contain additional domains that oxidize, circularize, or otherwise modify the peptide product and can even form hybrid enzymes with polyketide synthases.

NRPSs require activation through the attachment of phosphopantetheine groups to their peptidyl carrier domains by phosphopantetheine transferases (PPTases). Microorganisms utilize multiple PPTases for different groups of enzymes [9,10]. PPTases are also present in animals because they are required for the activation of acyl carrier proteins involved in fatty acid metabolism. In the springtail *Folsomia candida*, in which an NRPS was previously identified, only a single PPTase was found [11]. This PPTase was coregulated with the NRPS during stress, suggesting that a native arthropod enzyme was recruited to activate the NRPS obtained by horizontal gene transfer.

Other types of NRPSs are also known to exist in animals. These are the single-module Ebony [12] (in *Drosophila melanogaster* and other insects) and acyl CoA-synthetase family member 4 (ACSF4-U26) (e.g., in mammals) [13]. However, these enzymes use other domains, not the condensation domains found in the NRPSs investigated here [14]. The Ebony protein is involved in pigmentation and other processes [15]. ACSF4-U26 is a beta-alanine-activating enzyme that may be involved in post-translational modification [13].

While the diversity of bacterial and fungal NRPSs is staggering and receives a lot of attention, due to their great biotechnological potential, a comparative study of NRPSs in the animal kingdom has not yet been conducted. In this study, we analyzed 1059 sequenced animal genomes for NRPSs to map the distribution and abundance of metazoan NRPSs and provide insight into their evolutionary history. We identified candidate NRPS genes in 86 species from 7 different phyla. NRPSs appeared to be especially abundant in rotifers, where we found both NRPS–PKS and NRPS genes with many different predicted substrates. Finally, we describe a second potential case of beta-lactam biosynthesis capacity in an animal genome, the nematode *Plectus sambesii*.

## 2. Materials and Methods

### 2.1. NRPS Identification

We analyzed 1059 genomes from 19 animal phyla (Supplementary Table S1) for putative NRPS genes using antiSMASH software version 4.1.0 [16]. Both the bacterial and fungal versions of antiSMASH were used for the analysis. For each scaffold or contig containing a putative NRPS, we performed a check for contamination, since genome assemblies sometimes contain sequences from microorganisms. We extracted the scaffold sequences from the genomes and ran a BLASTN against the NCBI nonredundant database. The idea is that the putative NRPS may resemble microbial sequences but that (parts of) the surrounding area in the genome should be similar to metazoan sequences.

We manually analyzed the results and considered a scaffold as an animal sequence when at least one hit with an e-value <1 × 10^−10^ against another animal was found. When no such hit was found, but instead a hit against a microorganism with an e-value <1 × 10^−10^ was returned, we considered the scaffold potentially contaminated. When no hits below the threshold against any organism were found, we manually analyzed the scaffold by BLASTing predicted protein sequences from the scaffold against the NCBI nonredundant database. In some cases, the nature of the scaffold (microbial or metazoan) could not be reliably determined (usually when the scaffold was very short). These cases were noted as inconclusive and were not used for further analysis.

Since antiSMASH did not identify the NRPSs previously reported in Platyhelminthes [4] (probably due to their specific domain architecture) we used the *Schistosoma mansoni* NRPS (XP_018648700) to analyze other animal phyla for these types of NRPSs. We conducted a TBLASTN against the nr/nt database of NCBI and included each hit with an e-value <1 × 10^−10^ in our analysis. To visualize the distribution of the analyzed genomes and identified putative NRPSs, we extracted a phylogenetic tree of the animal phyla from the open tree of life version 13.4 [17] using the rotl package version 3.0.12 [18] in R 4.1.0 (R Core Team, 2021).

### 2.2. Phylogenetic Analyses

Our phylogenetic analysis of NRPSs in animals was wholly based on the adenylation domains; however, these domains are also found in other enzymes present in animals (e.g., in acyl-coenzyme A synthetases). To make sure that the identified putative NRPSs in animals were not from this group, we included other metazoan adenylate-forming enzymes in our analysis. The amino acid sequences of the adenylation domains identified by antiSMASH were extracted. A set of metazoan adenylate-forming enzymes (Supplementary Table S2) was extracted from UniProt [19] to be used as the outgroup. InterProScan [20] was used to predict the adenylation domains of the outgroup proteins as well as the proteins identified using the *S. mansoni* NRPS (Sm-NRPS), and these domain sequences were extracted.

Only the extracted adenylation domains ≥300 amino acids were used in the phylogenetic analysis. The adenylation domains of all animals except rotifers (which were used in a separate analysis) were aligned using Clustal Omega version 1.2.4 [21] using default settings. Columns with <70% coverage were trimmed using trimAl version 1.4.rev15 [22]. The resulting alignment was manually inspected and sequences with gaps over a large stretch of the alignment and sequences that contained Xs were removed before realigning and trimming. A phylogenetic tree was inferred by maximum likelihood using IQTREE version 2.2.3 [23] using the Q.pfam + F + R7 model with 1000 bootstrap replicates.

An ACVS-based phylogenetic tree was constructed using IQ-TREE version 2.0.6 [23]. The JTT + I + G4 model was used with 1000 bootstrap replicates. The IPNS-based phylogenetic tree was constructed using the LG + G4 model with 1000 bootstrap replicates. The cefEF-based phylogenetic was constructed using the WAG + I + G4 model with 1000 bootstrap replicates.

A phylogenetic analysis of rotifer sequences was performed using IQTREE version 2.0.6 [23] using the LG + F + R5 model with 1000 bootstrap replicates. The phylogenetic trees were visualized using iTOL version 6 [24].

### 2.3. NRPSs in Rotifers

Because so many putative NRPSs were identified in rotifers, we did a separate analysis for this group. The adenylation domains were extracted from these sequences and aligned to the adenylation domains of a selection of other rotifer adenylate-forming enzymes (Supplementary Table S2). These domains were aligned using Clustal Omega version 1.2.4 [21] using default settings, and the alignment was manually inspected. Using trimAl version 1.4.rev15 [22], columns with <70% coverage were trimmed. The substrate predictions (based on profile hidden Markov models) for the adenylation domains of putative rotifer NRPSs were extracted from the antiSMASH output.

### 2.4. Beta-Lactam Biosynthesis Genes in Plectus Sambesii

AntiSMASH identified a putative NRPS gene in the nematode *Plectus sambesii* that resembled L-δ-(α-aminoadipoyl)-L-cysteinyl-D-valine synthetase (ACVS) (approximately 50% identity on the amino acid level). ACVS catalyzes the first step in beta-lactam biosynthesis in bacteria and fungi and potentially also in springtails. Because this would represent another remarkable case (in addition to Collembola) of beta-lactam biosynthesis genes in animals, we did a separate analysis for these genes in nematodes. We identified other beta-lactam biosynthesis genes in *P. sambesii* using TBLASTN with a selection of beta-lactam biosynthesis genes from microorganisms (Supplementary Table S3). Genome regions with a hit below the threshold of 1 × 10^−10^ were translated to proteins and used in a BLASTP against the NCBI nonredundant database. When they had a lower e-value against a non-beta-lactam protein, they were excluded from further analysis. 

Phylogenetic trees of ACVS, IPNS and cefEF proteins from bacteria, fungi, springtails, and *P. sambesii* were inferred to see whether the nematode proteins form monophyletic clades with other animals. The proteins were aligned using Clustal Omega version 1.2.4 [21] with default settings, and the resulting alignments were manually adjusted (Supplementary File S1).

## 3. Results

### 3.1. The Distribution of NRPS Genes in Animals

**Figure 1:**
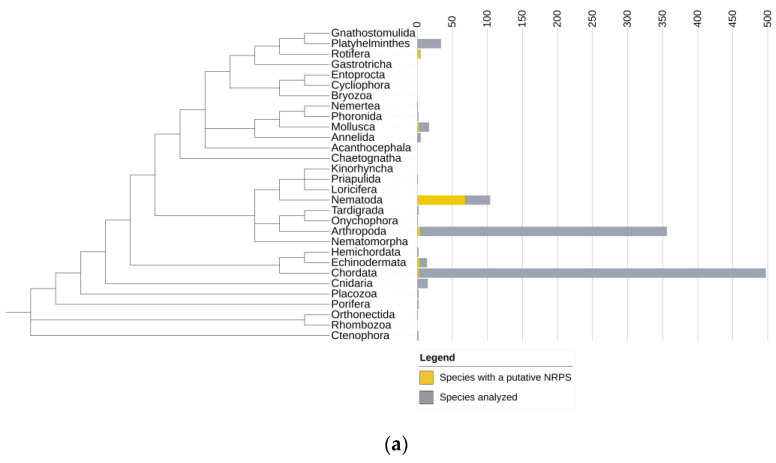

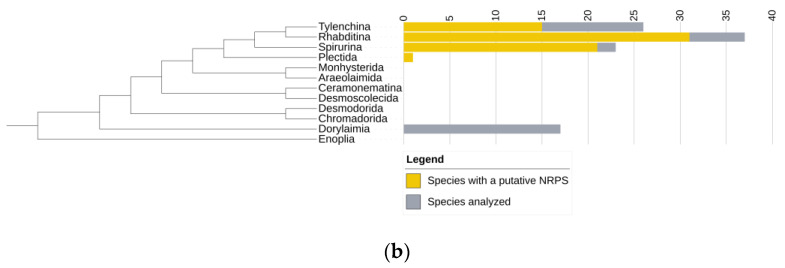
Distribution of identified putative nonribosomal peptide synthetases (NRPSs) across the animal kingdom. The sums of the yellow and grey bars represent the number of genomes analyzed. The yellow bars represent the number of genomes that contained at least one putative NRPS identified by antiSMASH. (**a**) NRPSs were scattered over the animal phyla; they were present in both early diverging phyla and phyla that evolved later. Most sequenced genomes were from Arthropoda, Chordata, and Nematoda. Putative NRPSs were identified in Nematoda, Rotifera, Arthropoda, Chordata, Phoronida, Mollusca and Echinodermata. NRPSs not recognized by antiSMASH (e.g. in Platyhelminthes) are not included here (**b**) Species from five nematode clades were analyzed in more detail. NRPSs were identified in Rhabditina, Tylenchina, Spirurina, and Plectida. No putative NRPSs were identified in Dorylaimia. The nematode phylogeny was based on [25].

Genomic data were available for 19 animal phyla, with most genomes representing Chordata, Nematoda, and Arthropoda. We identified NRPS clusters in over 8% of the 1059 animal genomes analyzed (Figure 1a, Supplementary Table S4). For each NRPS cluster identified by antiSMASH, we conducted a contamination check to analyze whether the putative NRPSs were truly part of the animal genome by evaluating whether the surrounding genes had best BLAST hits to other animals. AntiSMASH identified 772 putative NRPS clusters, of which 199 passed the contamination check and are likely true animal sequences (Supplementary Table S4). The occurrence of NRPSs showed a scattered distribution over metazoan phyla: Annelida, Arthropoda, Chordata, Cnidaria, Echinodermata, Hemichordata, Mollusca, Nematoda, Nemertea, Phoronida, Platyhelminthes, Priapulida, and Rotifera. Within the phylum Nematoda, genomes were available from Rhabditina, Tylenchina, Spirurina, Plectida, and Dorylaimia. NRPS and NRPS–polyketide synthases (NRPS–PKS) were identified in many species, but not in the early-diverging Dorylaimia (Figure 1b).

**Figure 2:**
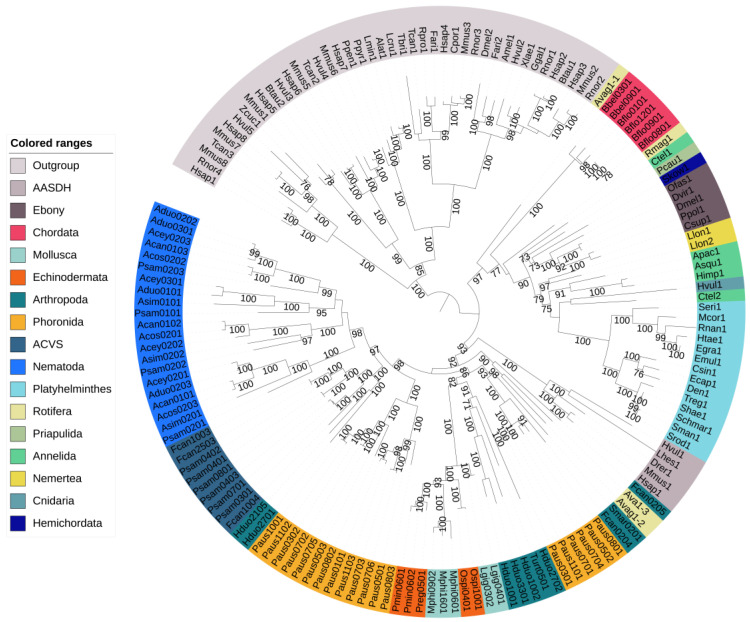
Phylogenetic tree of putative nonribosomal peptide synthetases and other adenylate-forming enzymes in animals. The outgroup proteins formed a monophyletic clade. Ebony clustered with NRPSs from Platyhelminthes and other proteins identified based on their similarity to Sm-NRPS. The chordata are also part of this clade. AASDH clustered with putative NRPSs identified by antiSMASH. NRPSs identified in Mollusca and Echinodermata were found in a single clade in the tree. The nematode NRPSs formed a monophyletic clade except for several *Plectus sambesii* adenylation domains, which clustered in the ACVS clade. Bootstrap support ≥70% is shown.

Figure 2 shows the phylogenetic relationships among all animal NRPS sequences. NRPSs from Platyhelminthes clustered with Ebony as expected. The A domains from Annelida, Priapulida, Hemichordata, Nemertea and Cnidaria identified using Sm-NRPS were also part of this clade (Figure 2 and Table S5). In this clade, we also found *Branchiostoma belcheri* and *Branchiostoma floridae* (Chordata). In contrast to Ebony proteins, the *Branchiostoma* proteins did appear to contain regular condensation domains. A rotifer NRPS that showed similarity to Sm-NRPS contained one adenylation domain that clustered with Ebony and two adenylation domains that clustered with NRPSs identified by antiSMASH.

The NRPSs in Nematoda formed a monophyletic clade except for some *Plectus sambesii* adenylation domains. This is discussed below under beta-lactam biosynthesis genes in *P. sambesii*. A tree with all nematode adenylation domains is available in Supplementary Figure S1. In the springtail *F. candida* (Arthropoda), we identified a second NRPS, in addition to the two copies of ACVS reported previously, on scaffold LNIX01000001.1. Furthermore, in another springtail, *Holacanthella duospinosa*, we also found an NRPS that was different from ACVS.

The NRPSs identified in Echinodermata (*Ophiothrix spiculata*, *Patiria miniate*, *Patiriella regularis*) and Mollusca (*Lottia gigantea*, *Modiolus philippinarum*) formed a monophyletic clade, even though the phyla themselves were not related at all (echinoderms are deuterostomes, molluscs are protostomes) (Figure 1a). It should be noted that all NRPSs in molluscs and echinoderms are located on short scaffolds, which makes it more difficult to achieve absolute certainty regarding the absence of contamination. Lastly, a clade of arthropod NRPSs consisting of *Tetranychus urticae* (Acari) and *Holacanthella duospinosa* (Collembola) was present in the tree.

### 3.2. Putative NRPSs Are Abundant in Rotifers

**Figure 3:**
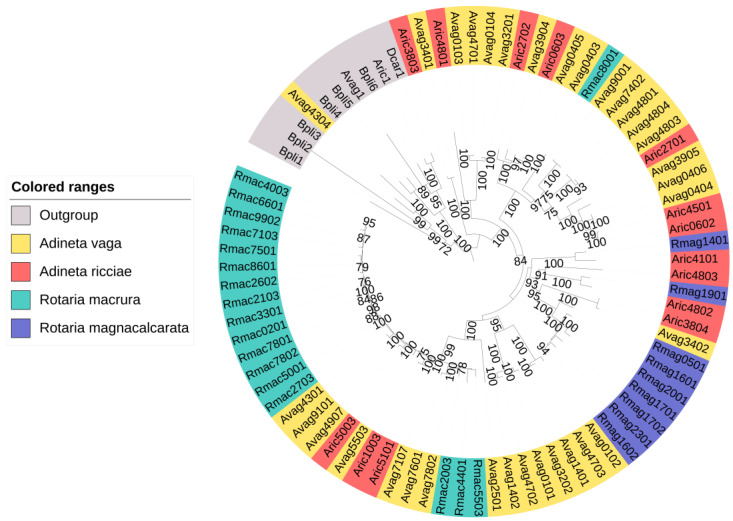
Phylogeny of the identified putative rotifer NRPSs and other rotifer adenylate-forming enzymes (outgroup). One of the putative NRPSs clustered with the outgroup proteins. The others formed separate clades. Bootstrap support ≥70% is shown.

**Figure 4:**
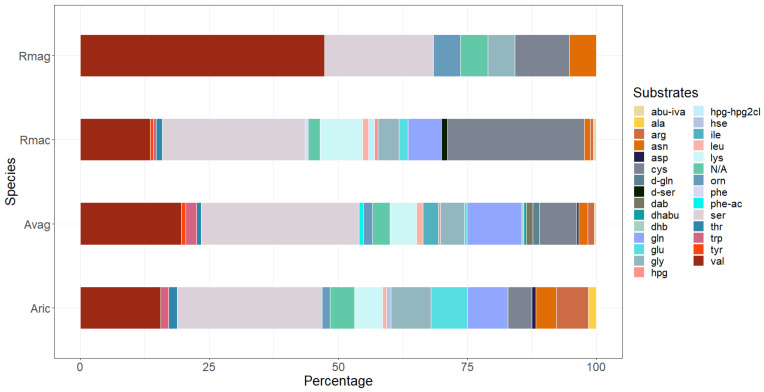
Substrate predictions for putative nonribosomal peptide synthetases (NRPSs) in rotifers. Rotifer NRPSs are predicted to use a wide variety of amino acid substrates including nonproteinogenic amino acids. Abbreviations: Rmag: *Rotaria magnacalcarata*; Rmac: *Rotaria macrura*; Avag: *Adineta vaga*; Aric: *Adineta ricciae*.

In rotifers, we identified up to 79 NRPS clusters per species (Table 1). One of these clustered with non-NRPS adenylating enzymes in the phylogenetic analysis (Figure 3). The putative NRPSs of the four rotifer species did not cluster in separate groups, but instead formed several clades together. We found both pure NRPSs and NRPS–PKS hybrids in rotifers. Moreover, large differences were observed between species in both the number of NRPS clusters and their predicted substrates (Table 1 and Figure 4). No NRPSs were identified in *Brachionus calyciflorus*, a rotifer that is classified outside the subclass Bdelloidea, to which the other four rotifers belong.

It should be noted that some rotifer NRPS clusters were rather short and might have in fact together formed a single NRPS. This would reduce the total number of NRPSs reported in Table 1. The smaller NRPS clusters are often located on small scaffolds that do not contain other genes. However, for *Adineta vaga*, a new chromosome-level assembly has been published [26], which contains most of the NRPS clusters that were identified here in the more fragmentary assemblies (Supplementary Table S4).

### 3.3. Beta-Lactam Biosynthesis Genes in P. sambesii

Several of the adenylation domains of the nematode *P. sambesii* clustered with springtail L-δ-(α-aminoadipoyl)-L-cysteinyl-D-valine synthetase (ACVS) (Figure 2). ACVS catalyzes the first step of beta-lactam antibiotic biosynthesis in microorganisms. Hence, we analyzed whether the genome of *P. sambesii* also contained other enzymes from the beta-lactam biosynthesis pathways. In addition to ACVS, we identified an isopenicillin N synthase (IPNS), which catalyzes the second step of the pathway that forms the beta-lactam ring and a deacetoxycephalosporin C synthase (*cefEF,* DAOC synhase).

**Figure 5:**
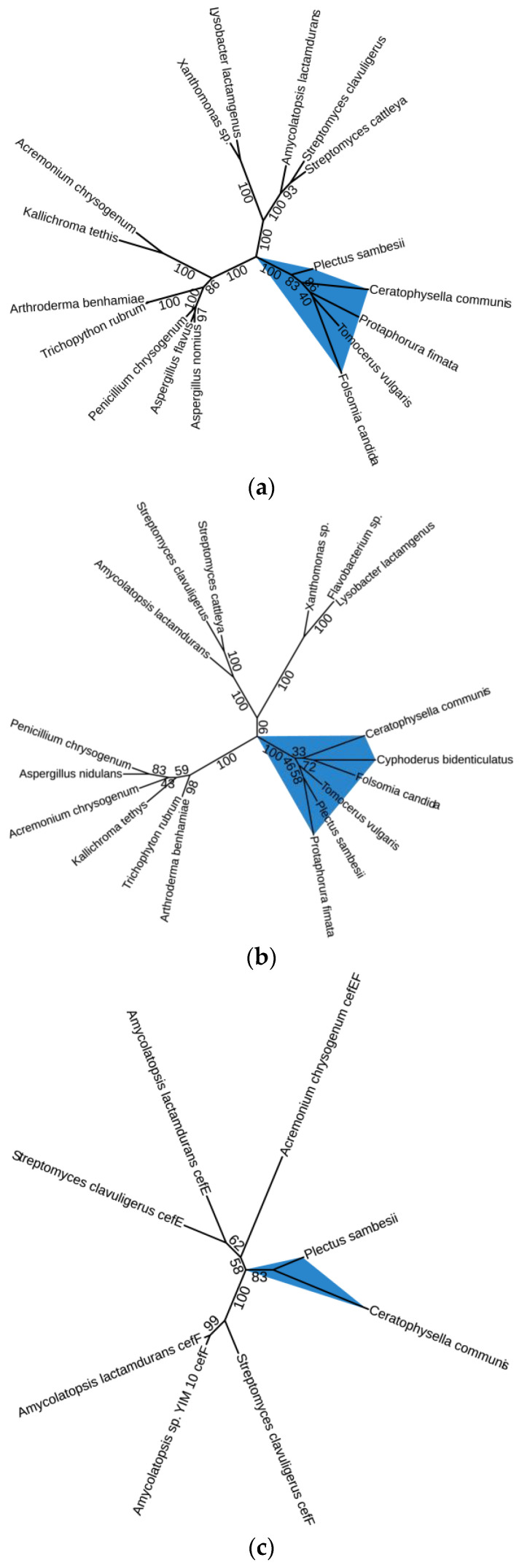
Phylogenetic analyses show that *Plectus sambesii* beta-lactam biosynthesis proteins form monophyletic groups with other animals (springtails). (**a**) L-δ-(α-aminoadipoyl)-L-cysteinyl-D-valine synthetase (ACVS), (**b**) isopenicillin N synthase (IPNS), and (**c**) deacetoxycephalosporin C synthase (cefEF).

The *P. sambesii* IPNS and deacetoxycephalosporin C synthase are found on the same scaffold (GenBank ID NIUQ01002120.1). The ACVS gene was scattered over multiple short scaffolds, and it could not be determined whether the assembly contained these scaffolds due to contamination (Supplementary Table S4). However, phylogenetic analyses were used to investigate whether the *P. sambesii* ACVS, IPNS, and DAOC synthase proteins would cluster within a clade of microorganisms or within the animal clade. All three proteins formed monophyletic clades with the previously identified ACVS, IPNS, and DAOC synthase proteins of springtails, indicating that this is an animal (nematode) ACVS (Figure 5).

**Figure 6:**
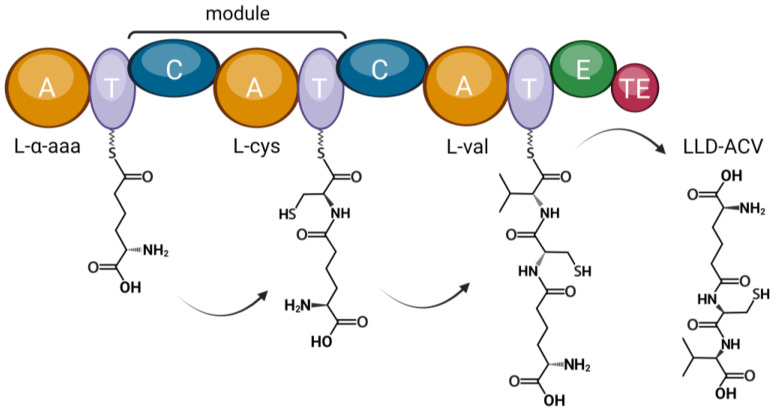
The domain architecture of ACVS. The structure and substrate predictions of a *Plectus sambesii* putative NRPS resemble that of bacterial, fungal and springtail ACVS. The predicted substrates based on the adenylation domains are: aminoadipic acid (aad), cysteine (cys), and valine (val). The final product is a tripeptide (LLD-ACV). The amino acid substrates are activated by the adenylation (A) domains and attached to phosphopantetheine groups that are bound to the thiolation (T) domains. Amide bonds between neighbouring substrates are formed by the condensation (C) domains. The epimerization (E) domain epimerizes L-valine while the thioesterase (TE) domain catalyzes the release of the peptide. Created with BioRender.com.

*P. sambesii* ACVS shares approximately 50% amino acid identity with *F. candida* ACVS, while *P. sambesii* IPNS has an identity percentage of approximately 65% to *F. candida* IPNS. The predicted substrates for the three adenylation domains of the *P. sambesii* protein are aminoadipic acid, cysteine, and valine, which are the expected substrates for all known ACVS proteins (Figure 6). *P. sambesii* was the only plectid nematode that was analyzed in this study.

## 4. Discussion

This study is the first kingdom-wide analysis of NRPSs in animals. We found that NRPSs are widespread in animals and in some cases very abundant. Over 8% of species and 30% of animal phyla that we analyzed contained NRPSs. For some phyla, we analyzed only a small number of genomes. Yet, in some of these phyla we still found putative NRPSs, indicating they may be abundant in these clades. Their presence appeared scattered over the animal tree as we found them in Chordata, Mollusca, Echinodermata, Phoronida, Arthropoda, Nematoda, and Rotifera.

The function and evolutionary history of NRPSs in animals is still not clear. Previously, it has been suggested that some of these genes may be the result of horizontal gene transfer events from bacteria to animals [1,6]. An analysis of an NRPS–PKS of *C. elegans* showed that based on its polyketide synthase domains (AT, KR and KS), it was most likely derived from an animal fatty acid synthase [5]. However, a phylogenetic analysis of its TE domain showed that it formed a group with bacterial NRPS–PKS TE domains [2].

If NRPSs were acquired by animals through horizontal gene transfer, then we might expect to find them in only few lineages, or at least restricted to certain clades, unless the transfer event happened deep in the Metazoa. What we observe is a scattered distribution over many animal phyla. This suggests that, if NRPSs were acquired through horizontal gene transfer, then this must have happened early in the evolution of the clade, maybe even at its origin. The scattered distribution would then be the result of gene loss.

Why rotifers, nematodes, and springtails are among the animal clades with the highest prevalence of NRPSs remains unclear. A property common to all three animal groups is the ability of some species to undergo anhydrobiosis, a phenomenon best investigated in tardigrades. The extreme dehydration during anhydrobiosis is known to be accompanied by DNA degradation [27,28,29,30,31,32]. Reassembly of the genome during rehydration could predispose anhydrobiotic animals to HGT. However, in the two species of tardigrade, we found no NRPS genes. Several NRPSs were found in phoronids (horseshoe worms, a small phylum of lophophorate marine invertebrates related to bryozoans), but what aspect of their ecology would make them prone to recruit NRPSs is another riddle.

NRPSs are particularly abundant in rotifers. Both their domain architecture and substrate specificity are highly variable. Rotifers exert the highest rate of horizontal gene transfer found to date in animals (approximately 7.5%) [30], and many horizontally transferred genes have been acquired recently [33]. Interestingly, we found no NRPSs in the only non-bdelloid species in our analysis. Hence, if NRPSs in rotifers have been acquired through horizontal gene transfer, then they may have been (1) transferred after the divergence of bdelloid rotifers, (2) transferred earlier in rotifer evolution but subsequently lost in some species, or (3) only one or a few NRPSs are ancestral, and they were lost in some species but expanded in bdelloids. As more non-bdelloid genomes are sequenced, it will be interesting to see if they contain NRPS genes.

The identified metazoan NRPSs did not cluster with adenylate-forming enzymes in the outgroup except for Ebony and AASDH. In fungi, something similar was observed as aminoadipate reductases grouped with NRPSs, suggesting they are more similar to NRPSs than to other adenylate-forming enzymes [34]. We found that *Branchiostoma* putative NRPSs grouped with Ebony, indicating that these proteins may have evolved from a common ancestral gene. However, in the identified *Branchiostoma* sequences, we found condensation domains that were not present in Ebony.

Shou et al. (2016) found that nematodes utilize a multimodule NRPS and a multimodule NRPS–PKS to produce nemamide, a compound that promotes larval survival [2]. Homologs of these genes are present in more than twenty species of nematodes. In addition to the species previously identified to contain an NRPS, we identified 38 other species with NRPS genes. In most nematodes, we found an NRPS and an NRPS–PKS hybrid. Nematode NRPSs form a monophyletic group, suggesting a possible single origin and subsequent expansion into two types: a hybrid and a pure NRPS. Due to a lack of genomes from several clades of nematodes in our dataset, it was not possible to completely assess which nematode clades contained NRPSs. However, these genes appeared to be absent from Dorylaimia, suggesting that the NRPSs that produce nemamide could be exclusive to nematode clades that diverged more recently.

Beta-lactam antibiotics are produced by a subset of fungal and bacterial clades. The most famous beta-lactam is penicillin, which is produced by the fungus *Penicillium chrysogenum*. Recently, we identified the first beta-lactam biosynthesis pathway in an animal [3]. The springtail *F. candida* contains a unique combination of four beta-lactam biosynthesis genes, while other springtails contain different sets of beta-lactam genes [6]. In our previous analysis, we did not find beta-lactam genes in non-springtail animals, including its closest relatives Diplura and Protura. Here, we showed that beta-lactam biosynthesis genes are also present in the plectid nematode *P. sambesii*. These genes appeared to be limited to a small clade of nematodes since we did not find them in Rhabditina or Dorylaimia. Since only a single plectid genome was available, it is currently unclear how prevalent they are. It is interesting to observe that the combination of beta-lactam genes in *P. sambesii* was identical to the combination we found in several springtail species [6]. It was distinct from the beta-lactam gene clusters of fungi and bacteria. The first two genes that catalyze the formation of the core penem structure appeared to be included. This core structure involves a heterobicyclic ring, which is central to the antimicrobial activity of the beta-lactam compound [35].

It has been suggested that the beta-lactam biosynthesis pathway of bacteria may have been horizontally transferred from *Streptomyces* to fungi [36]. Subsequently, fungi may have recruited native enzymes to produce new beta-lactam compounds. For example, in the case of penicillin production—which is restricted to fungi—the acyltransferase that catalyzes the final step in the biosynthesis of penicillin appears to be derived from a native fungal gene [37]. Hence, it is possible that springtails and nematodes have also added native enzymes to their beta-lactam biosynthesis pathways.

Recent reports have shown that the diversity of secondary metabolites in animals may be much higher than thought earlier [38]. While previously only a limited number of NRPS genes were identified in animals, here we demonstrated that they are present in over 30% of the animal phyla that were analyzed. NRPSs are especially abundant in rotifers, where we found up to 79 clusters per species. Metazoan NRPSs showed a variety of domain architectures including NRPS–PKS hybrids. The data provided may open up a largely unexplored field of research: the functional significance of NRPS genes in animals. Such NRPSs could potentially be used for designing biotechnological products such as novel beta-lactam compounds.

## 5. Conclusions

Many more animal genomes than previously assumed contain nonribosomal peptide synthetase genes. The capacity of animals to synthesize secondary compounds playing a role in signaling, antibiotic synthesis, and other functions might be much greater than thought before. This could also lead to discovery of new NRPSs with biotechnological applications. They are scattered across the metazoan tree and some clades contain many more NRPSs than others. These genes are often assumed to have entered animal genomes by horizontal gene transfer from bacteria, but since they are not restricted to specific lineages, it seems unlikely that all of them represent HGT events, although some do for sure. In addition, it is unclear which ecological traits predispose an animal to recruit these genes. A more detailed analysis, concentrated on specific lineages, could reveal the underlying mechanisms.

## Figures and Tables

**Table 1:** Nonribosomal peptide synthetases (NRPSs) and hybrid nonribosomal peptide synthetase–polyketide synthases (NRPS–PKSs) in rotifers. The bdelloid rotifers from the genera *Adineta* and *Rotaria* contained many NRPSs, while they appeared to be completely absent in the genome of the non-bdelloid *Brachionus calyciflorus*.

Species	NRPS	NRPS–PKS
*Adineta ricciae*	46	2
*Adineta vaga*	69	5
*Rotaria macrura*	79	0
*Rotaria magnacalcarata*	12	1
*Brachionus calyciflorus*	0	0

## Data Availability

The analyzed genomes are publicly available through https://www.ncbi.nlm.nih.gov/ (accessed 2021–2023). Other datasets are available as Supplementary Files.

## Supplementary Materials

The following supporting information can be downloaded at: https://www.mdpi.com/article/10.3390/genes14091741/s1.
