# Supplementary material for: Nonribosomal Peptide Synthetases in Animals
Source: Genes (Basel). 2023 Aug 30;14(9):1741. doi: 10.3390/genes14091741 (PMC10531068; doi:10.3390/genes14091741)
Supplement: Supplementary file 1 [file genes-14-01741-s001.zip › Supplementary Table S3 and Figure S1.pdf]

Supplementary Table S3. The beta-lactam biosynthesis proteins that were used to search for beta-lactam genes in the genome of the nematode *Plectus sambesii*. Abbreviations: IPNS: isopenicillin N synthase; IPN:AT: isopenicillin N N-acyltransferase; DAOC: deacetoxycephalosporin C; DAC deacetylcephalosporin.

UniProt ID	Protein	Gene	Species
P18549	IPNS	pcbC	*Streptomyces clavuligerus*
P15802	IPN:AT	penDE	*Penicillium chrysogenum*
P18549	IPN: epimerase	cefD	*Streptomyces clavuligerus*
P18548	DAOC synthase	cefE	*Streptomyces clavuligerus*
P42220	DAOC hydroxylase	cefF	*Streptomyces clavuligerus*
P39058	DAC acetyltransferase	cefG	*Acremonium chrysogenum*
B5GZH3	3'-hydroxymethylcephem-O-carbamoyltransferase	cmcH	*Streptomyces clavuligerus*
NA[1]	*O*-carbamoyl DAC	cmcI	*Folsomia candida*
NA[2]	7-alpha-cephem-methoxylase	cmcJ	*Folsomia candida*

[1] Protein sequence:
MEPSTTNESKDLVEGNFVDLHRLRGLGDDPVYYPPILEDRPRLWPLDKWSSAPRDLGYDNFATEHWKGL
RLLKDPETQSVYHNILWEIKPKTIIELGVYSGGSLVWFRDLTKAFKFPSRLIGIDIDLSRCQIPEGEMDMISLH
QADCNNPESFAFLKDNVEHPILFIDDAHCNTFNVIKYAVNNFLKVGDFVMIEDTMGMWGRYSPKHLKS
HLASFKDVMALDLLYSNVPCQLKDGVFQVIKSNN

[2] Protein sequence:
MIKIKPGRDVQATINYSRSSTTEKWFIDTISCPNGNKITNAENDPISTTIHDLRGVEHNFSLDKNGFQAIFSP
TSVPSNTLLSGGDVLKTVYYPEVEKLLMEVTGADKAVAFDHTIRQSQVNSETWLHRLPVMRAHVDQTPK
SAWGRIALHSPEVQSFYRFQIINVWRPIVNIVYDYPLTMADFRSLNLHVDLMPTDLRYPEVWVKDKETYSV
KWNRSHAWYYWSCMTPDEVLMAKCYDSASQRLAEAYPLPLERGNRNLYCSESVAGLAPHTAFYDEKAS
MKGSGRKSIEIRTLVFYK

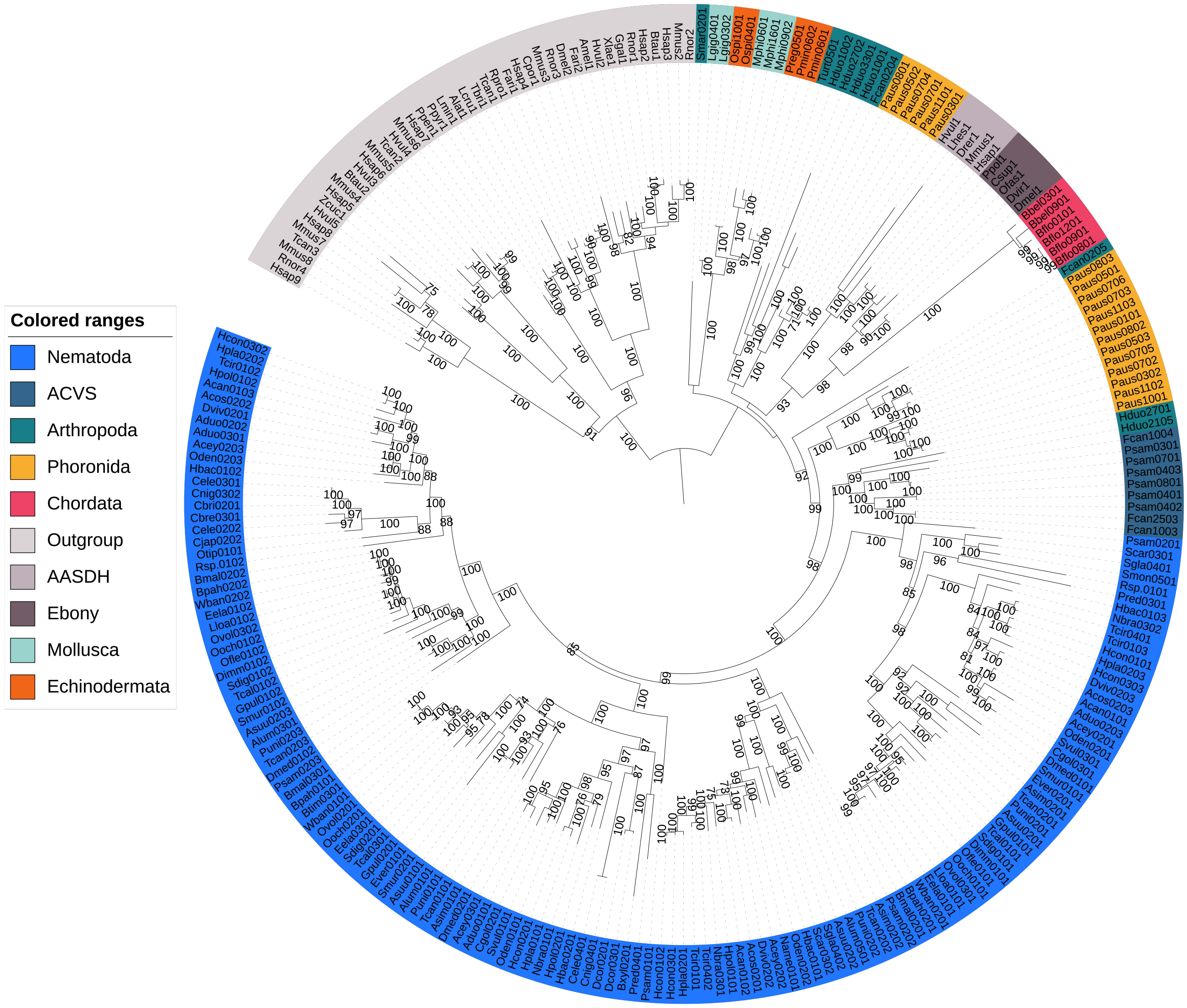

Supplementary Figure S1. A phylogenetic tree of identified putative NRPS adenylation domains in animals including all nematode sequences. The nematodes form a monophyletic clade except for several *Plectus sambesii* sequences that cluster with ACVS proteins of the springtail *F. candida*. Compared to Figure 2, the Mollusca, Echinodermata and a group of Arthropoda have moved and diverge before AASDH and Ebony (but with low support). Bootstrap support >70% is shown. The adenylation domains of outgroup proteins (Supplementary Table S2) and putative NRPSs were aligned using Clustal Omega version 1.2.4 and columns with <70% coverage were trimmed. The phylogenetic tree was inferred with IQTREE version 2.0.6 (Minh et al., 2020) using the LG+F+R8 model with 1000 bootstrap replicates.
